# Supplementary material for: LncRNA GAS5 inhibits microglial M2 polarization and exacerbates demyelination
Source: EMBO Rep. 2017 Aug 14;18(10):1801–16. doi: 10.15252/embr.201643668 (PMC5623836; doi:10.15252/embr.201643668)
Supplement: Supplementary file 1 — Appendix [file EMBR-18-0-s001.pdf]

APPENDIX

Long Noncoding RNA GAS5 Inhibits Microglial M2 Polarization and Exacerbates EAE

Dingya Sun#, Zhongwang Yu#, Xue Fang#, Mingdong Liu, Yingyan Pu, Qi Shao, Dan Wang, Xiaolin Zhao, Aijun Huang, Zhenghua Xiang, Chao Zhao, Robin J.M. Franklin, Li Cao* and Cheng He*

Contents

Appendix table S1: Sequence of FISH probes (Page 1)
Appendix table S2: QPCR primers for mouse genes (Page 2)
Appendix table S3: QPCR primers for human genes (Page 2)
Appendix figure S1. The lentivirus-mediated silencing of GAS5 and EZH2 in microglia (Page 3)
Appendix figure S2. ChIP analysis of microglia with the anti-H3K4me3 antibody (Page 3)

Appendix table S1.

**The sequence of FISH probe for mouse GAS5:**	**The sequence of FISH probe for human GAS5:**
AGCCTTTCGGAGCTGTGCGGCATTCTGAGCAGGAAT<br>GGCAGTGTGGACCTCTGTGATGGGACATCTTGTGGG<br>ATCTCACAGCCAGTTCTGTGGCAAAGGAGGATGAA<br>GGCTTACGAGGACTCGTCAGGAAGCTGGATAACAG<br>AGCGAGCGCAATGTGCTAGAATAGAAGACCAGAAA<br>ATGAAATGGTGGAGTTTGAGGCTGGATAGACAGTTT<br>GAAAGTTAACTGGTTGCATGCTTGTTCATTTGGCTG<br>GCTTGCTTGGGTACAAATAATGGTTTGAATAAAGAA<br>AGGTATTAATGGGTCACCTCAAGTGAAGGCACTGCA<br>AACACAATGATTGGTCATTCTGAATTTCCGGTCCTTC<br>ATTCTGAATTTCAAAGGCTCCTGTGACAAGTGGACA<br>TGCAGTGACTGCACCTTTGTTTCTGAGGTGCCTGGA<br>TGGAGGCTCAAATAGAAGATGGTGTCAGATATATTG<br>TGTTAAAATTTTACCATTAAAGTGTATTATAACATG	GCATTAGACAGAAAGCTGGAAGTTGAAATGGTGGA<br>GTCCAACTTGCCTGGACCAGCTTAATGGTTCTGCTC<br>CTGGTAACGTTTTTATCCATGGATGACTTGCTTGGGT<br>AAGGACATGAAGACAGTTCCTGTCATACCTTTTAAA<br>GGTATGGAGAGTCGGCTTGACTACACTGTGTGGAG<br>CAAGTTTTAAAGAAGCAAAGGACTCAGAATTCATG<br>ATTGAAGAAATGCAGGCAGACCTGTTATCCTAAACT<br>AGGGTTTTTAATGACCACAACAAGCAAGCATGCAG<br>CTTACTGCTTGAAAGGGTCTTGCCTCACCCAAGCTA<br>GAGTGCAGTGGCCTTTGAAGCTTACTACAGCCTCAA<br>ACTTCTGGGCTCAAGTGATCCTCAGCCTCCCAGTGG<br>TCTTTGTAGACTGCCTGATGGAGTCTCATGGCACAA<br>GAAGATTAAAACAGTGTCTCCAATTTTAATAAATTTT<br>TGCAATCCA

Appendix table S2. QPCR primers for mouse genes:

TNF-α	F	GCCTCCCTCTCATCAGTTCT
	R	ACTTGGTGGTTTGCTACGAC
IL-1β	F	TTCAGGCAGGCAGTATCA
	R	GTCACACACCAGCAGGTTAT
Ym-1	F	TACTCCTCAGAACCGTCAGAT
	R	CATTTCCTTCACCAGAACAC
Fizz1	F	ATGCCAACTTTGAATAGGATG
	R	CTTGACCTTATTCTCCACGAT
CD206	F	AAGATCTCATGGGCAACATCG
	R	CTTGCCAGGATAGTAAATGAGCAAT
IGF-1	F	ATGCTCTTCAGTTCGTGTGT
	R	GGCTGCTTTTGTAGGCTTC
GAPDH	F	TCAACGACCCCTTCATTGACC
	R	CTTCCCGTTGATGACAAGCTTC
IRF4	F	ATTGTTTAAAGGCAAGTTCCGAGA
	R	CTCGACCAATTCCTCAAAGTCA
GR	F	CGCTAACATTAATTTCCGTGTG
	R	GACTAGGTGCTCTATACCAGT
GAS5	F	GGCAAATGAGCACTAAAG
	R	CACCCACTCCTCTATCTACA

Appendix table S3. QPCR primers for human genes:

TNF-α	F	GCATCGCCGTCTCCTACCAG
	R	CGCTGAGTCGGTCACCCTTC
IL-1β	F	GCTACGAATCTCCGACCAC
	R	ACATAAGCCTCGTTATCCCA
iNOS	F	TCCCACCTGACCTTGTGCTT
	R	AGGGCGTACCACTTTAGCTC
Arg-1	F	AAGCCTATTGACTACCTTAACCC
	R	ATGCCATTAACTATGAGATTTATATCGG
CD206	F	ATACCTGCGACAGTAAACGA
	R	TTGCAGTATGTCTCCGCTTC
IGF-1	F	CACATCACATCCTCTTCG
	R	CTGGAGCCGTACCCTGTG
GAPDH	F	CTCTGACTTCAACAGCGACACC
	R	CTGTTGCTGTAGCCAAATTCGTT
GAS5	F	CTCTGGATAGCACCTTATGGAC
	R	CTTCCAGCTTTCTGTCTAATGCC

Appendix figure S1. The lentivirus-mediated silencing of GAS5 and EZH2 in microglia.

Quantitative PCR analysis of GAS5 (a) and EZH2 (b) in microglia transduced with the GAS5i or EZH2i lentivirus versus the control, n = 3 experiments. ***$P < 0.001$ versus control (Student's *t*-test). Data are shown as mean ± SD.

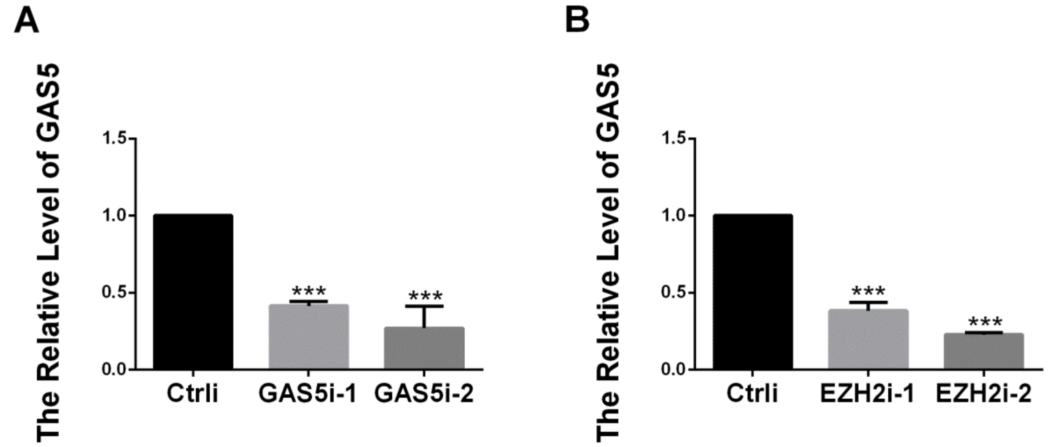

Appendix figure S2. ChIP analysis of microglia with the anti-H3K4me3 antibody.

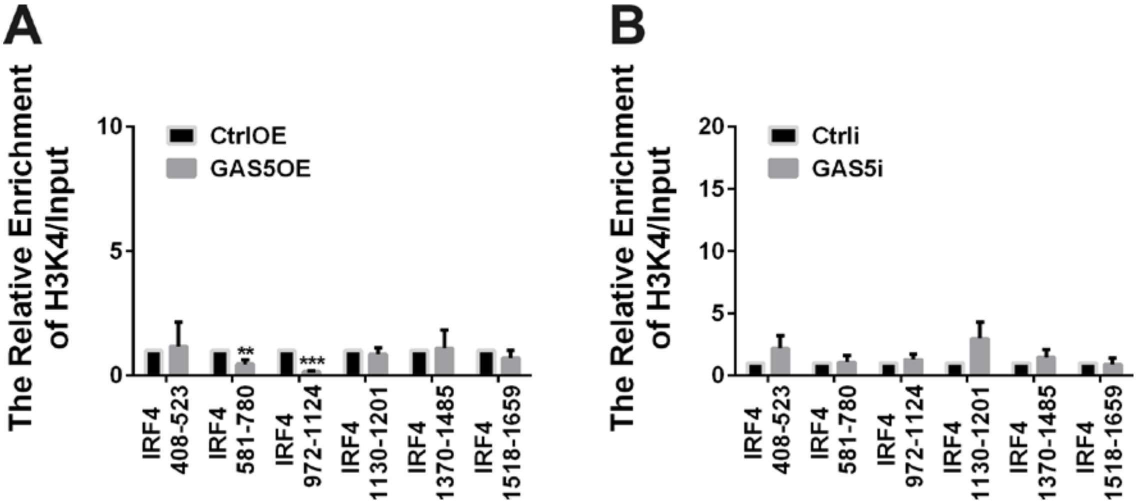

ChIP analysis of microglia transduced with the CtrlOE/GAS5OE (**a**) or Ctrli/GAS5i (**b**) lentivirus on the IRF4 promoter regions using the anti-H3K4me3 antibody, n = 3 experiments. **$p < 0.01$, ***$P < 0.001$ versus control (Student's *t*-test). Data are shown as the mean ± SD.
