# Supplementary material for: LncRNA GAS5 inhibits microglial M2 polarization and exacerbates demyelination
Source: EMBO Rep. 2017 Aug 14;18(10):1801–16. doi: 10.15252/embr.201643668 (PMC5623836; doi:10.15252/embr.201643668)
Supplement: Supplementary file 2 — Expanded View Figures PDF [file EMBR-18-0-s002.pdf]

Expanded View Figures

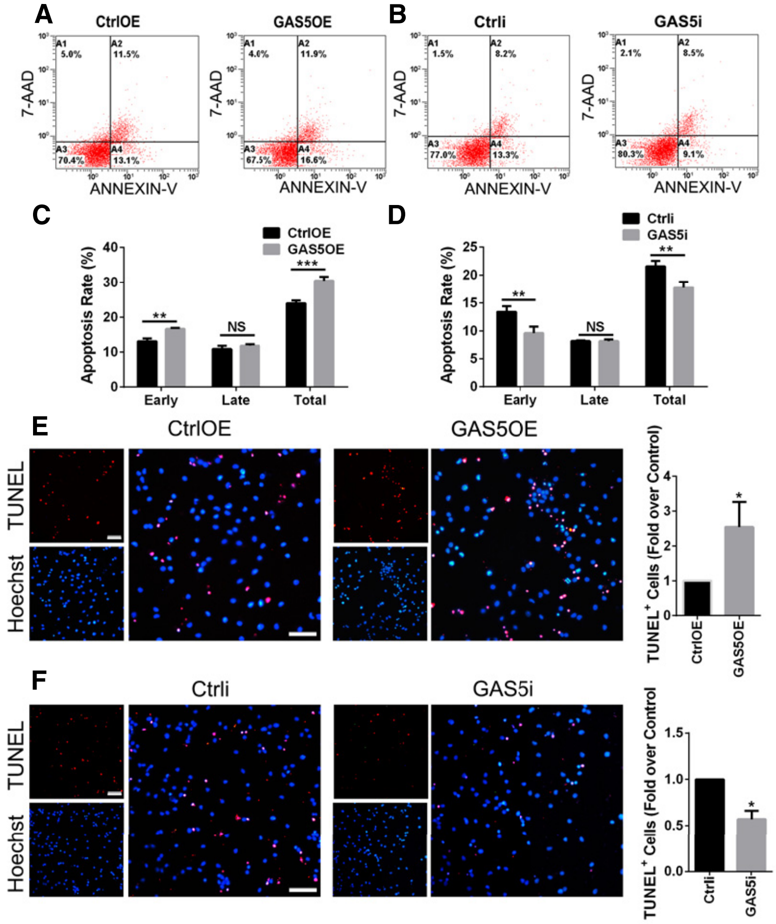

Figure EV1. GAS5-modified microglia affect OPC survival.

A, B Representative flow cytometry analysis after Annexin-V/7-AAD staining in OPCs incubated with conditioned medium from MG^{GAS5OE} (A) or MG^{GAS5i} (B) for 24 h versus the control.

C, D Apoptosis rate of the OPCs in (C) and (D) respectively, $n = 3$ experiments.

E, F TUNEL analysis of apoptosis in OPCs incubated with conditioned medium from MG^{GAS5OE} (E) or MG^{GAS5i} (F) for 24 h versus the control, $n = 3$ experiments. The ratio of $TUNEL^{+}$/$Hoechst^{+}$ cells was calculated and compared between groups. Scale bars = 50 μm.

Data information: $*P < 0.05$, $**P < 0.01$, $***P < 0.001$ versus control (Student's t-test). Data are shown as the mean ± SD.

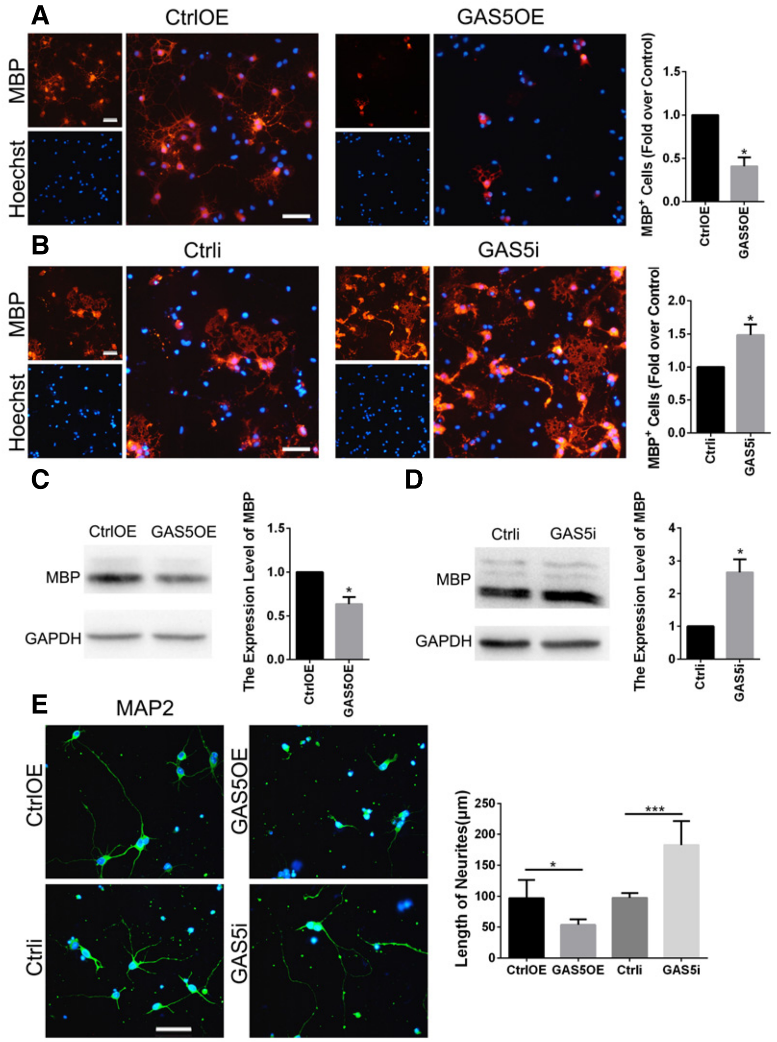

Figure EV2. GAS5-modified microglia affect OPC differentiation and neurite growth.

A, B Anti-MBP immunocytofluorescence of OPCs incubated with conditioned medium from MG^{GAS5OE} (A) or MG^{GAS5i} (B) for 48 h versus the control. Scale bars = 50 μm. The ratio of MBP^+/$Hoechst^+$ cells was calculated and compared between groups, n = 3 experiments.

C, D Western blotting analysis of MBP in OPCs incubated with conditioned medium from MG^{GAS5OE} (C) or MG^{GAS5i} (D) for 48 h versus the control, n = 3 experiments.

E Anti-MAP2 immunocytofluorescence of neurons incubated with conditioned medium from MG^{GAS5OE} or MG^{GAS5i} for 48 h versus the control, $n \geq 4$ experiments. Scale bar = 50 μm.

Data information: *$P < 0.05$, ***$P < 0.001$ versus control (Student's t-test). Data are shown as the mean ± SD.
Source data are available online for this figure.

 © 2017 The Authors

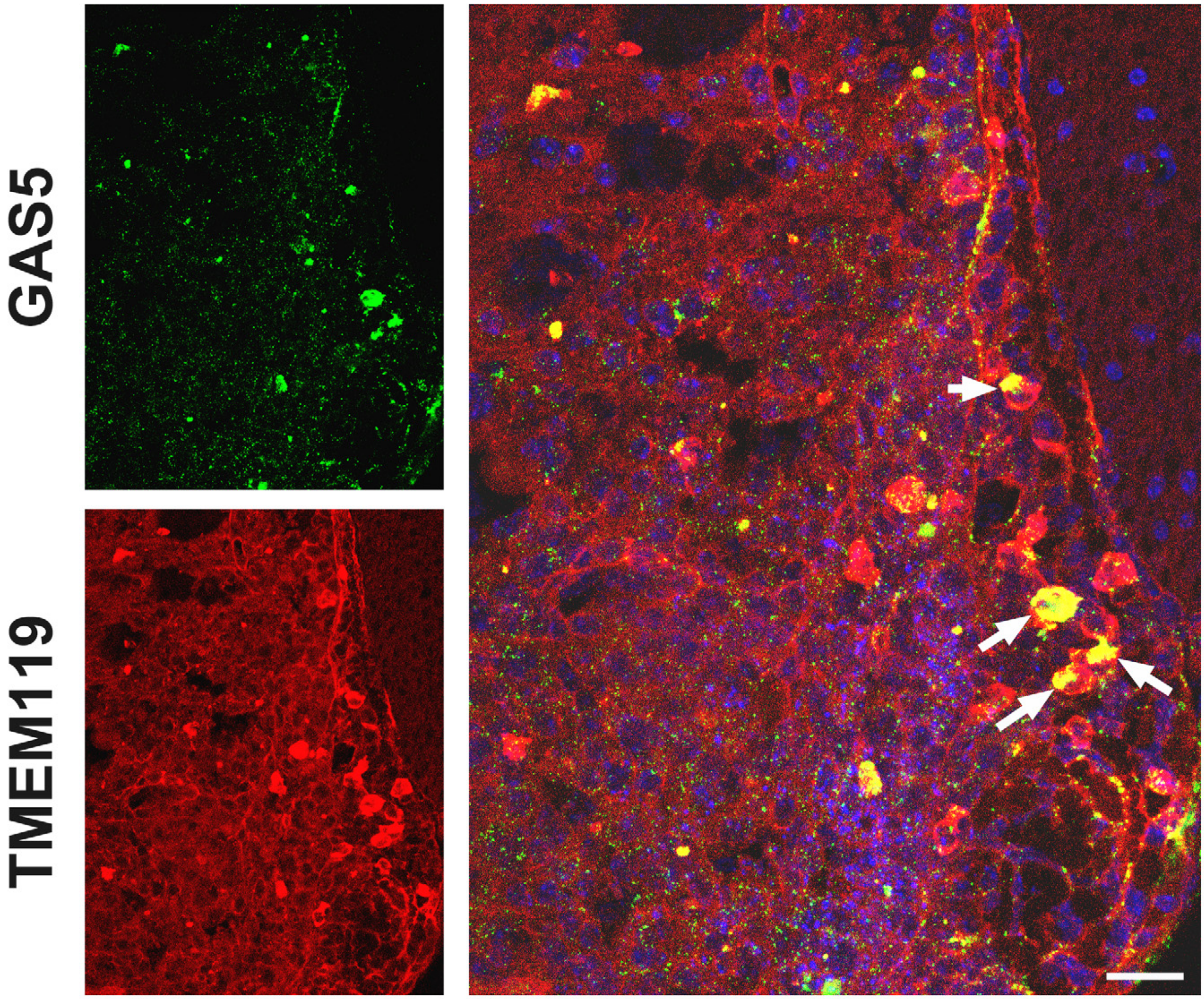

Figure EV3. GAS5 is expressed in the microglia of EAE.
Representative FISH analysis of GAS5 (green) co-stained with an anti-TMEM119 antibody (red) in spinal cord sections from EAE mice at 30 dpi. Arrows indicate GAS5$^+$TMEM119$^+$ cells. Scale bars = 25 μm.

© 2017 The Authors

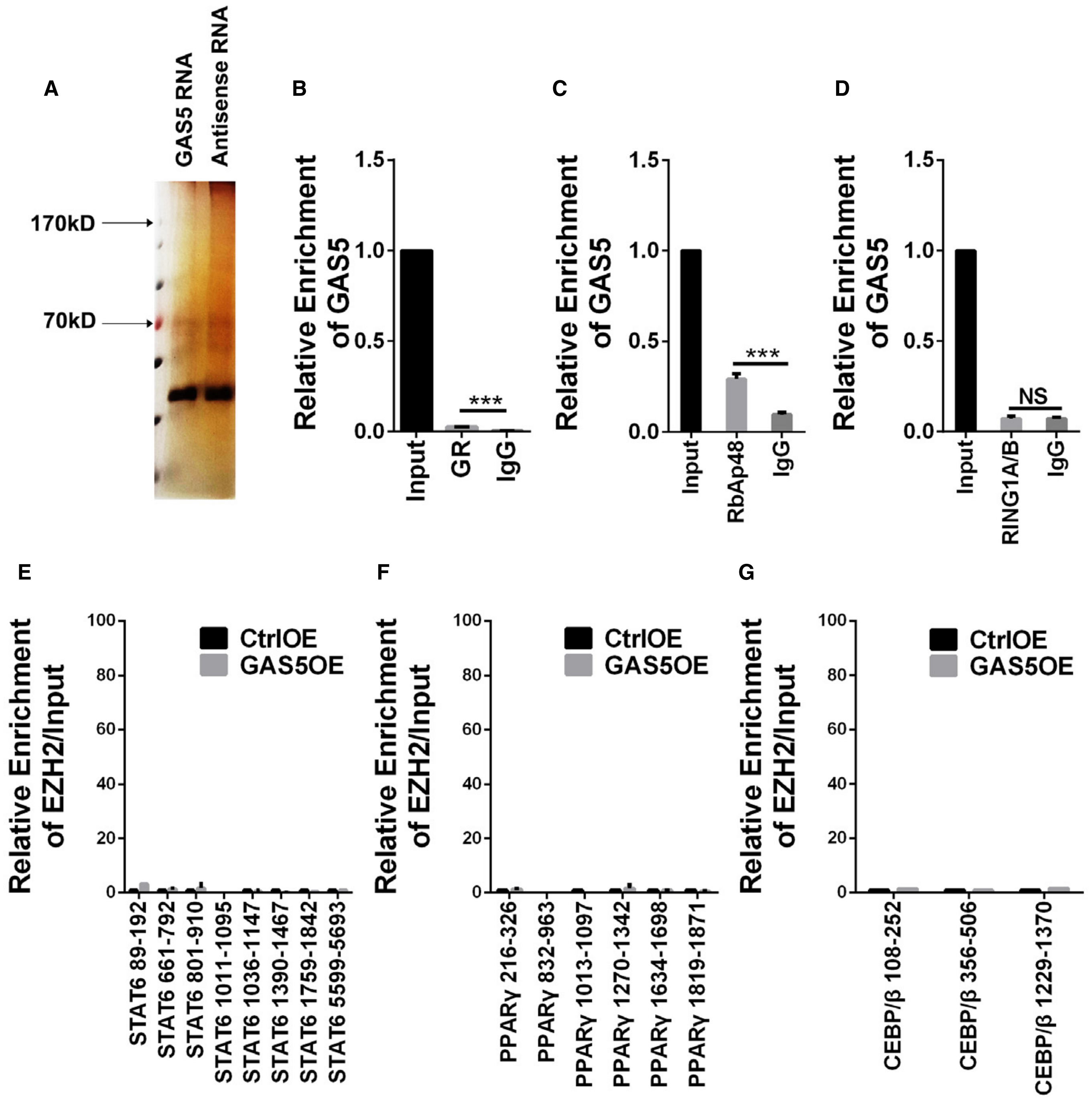

Figure EV4. Analysis of the mechanism regulating microglial polarization by GAS5.

A Silver staining to identify specific binding partners of GAS5 after RNA pull-down experiment.

B RNA IP analysis between GR and GAS5. *N* = 3 experiments.

C RNA IP analysis between RbAp48 and GAS5. *N* = 3 experiments.

D RNA IP analysis between RING1A/B and GAS5. *N* = 3 experiments.

E–G ChIP analysis of microglia transduced with the CtrlOE or GAS5OE lentivirus. The promoter regions of STAT6 (E), PPARγ (F) and CEBP/β (G) were detected in MG^{GAS5OE} versus the control using the anti-EZH2 antibody.

Data information: ***$P < 0.001$ (B–D, Student's *t*-test). Data are shown as the mean ± SD.

 © 2017 The Authors

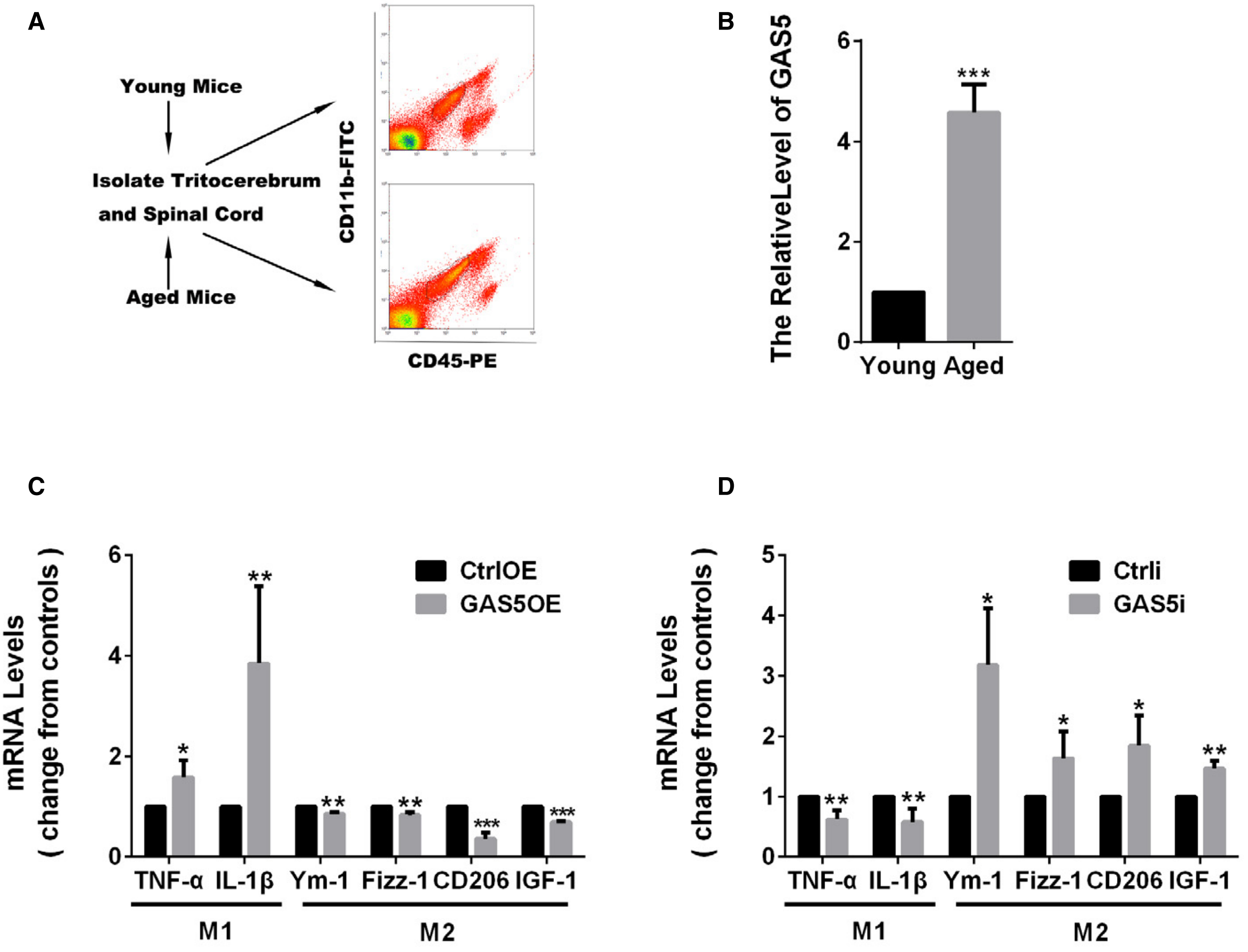

Figure EV5. GAS5 is highly expressed in aged microglia and regulates the inflammatory response of monocytes.

A A schematic map of microglial isolation from young (6 weeks) and aged mice (> 15 months).

B Quantitative PCR analysis of GAS5 in microglia from aged mice versus young mice, n = 3 experiments.

C, D Quantitative PCR analysis of M1 and M2 markers in monocytes transduced with the GAS5OE (C) or GAS5i (D) lentivirus vectors versus the control, $n \geq 3$ experiments.

Data information: *$P < 0.05$, **$P < 0.01$, ***$P < 0.001$ (B–D, Student's t-test). Data are shown as the mean ± SD.

© 2017 The Authors
