# Supplementary figures and images for: LncRNA GAS5 inhibits microglial M2 polarization and exacerbates demyelination
Source: EMBO Rep. 2017 Aug 14;18(10):1801–16. doi: 10.15252/embr.201643668 (PMC5623836; doi:10.15252/embr.201643668)

**Figure EV2C**

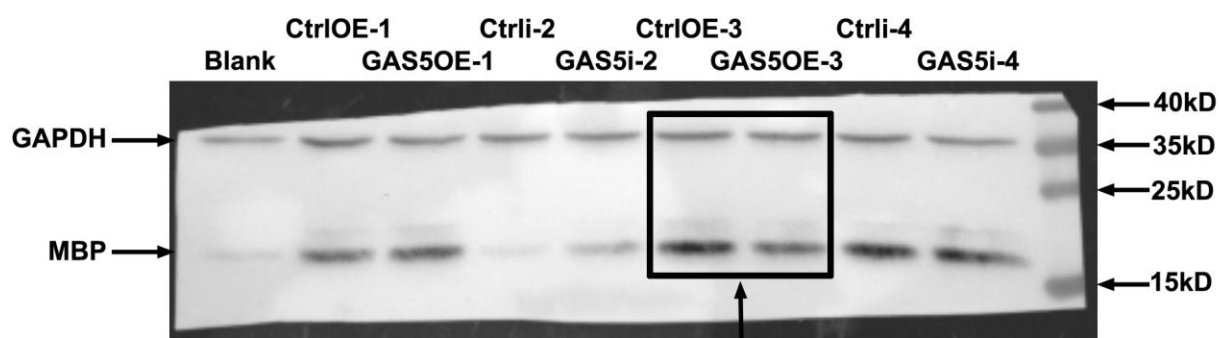

**Figure EV2C**

**Figure EV2D**

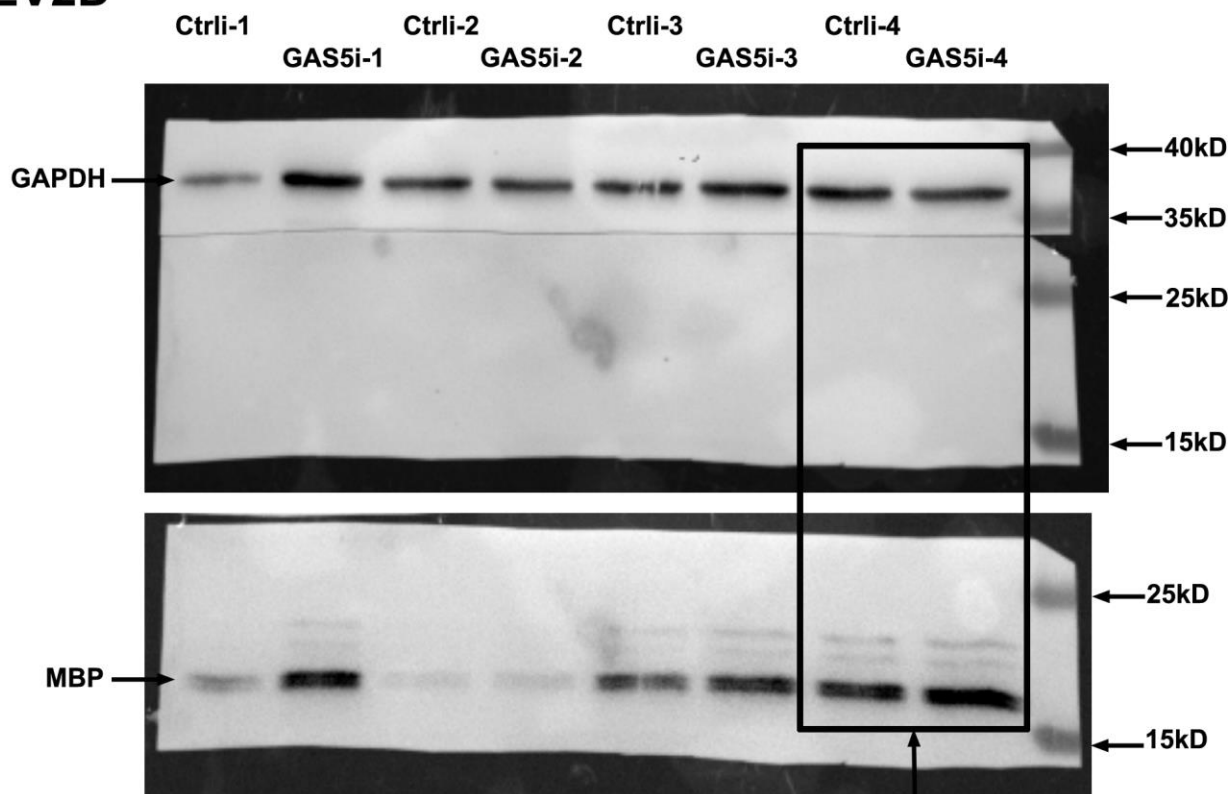

**Figure EV2D**

Supplement: Supplementary file 3 — Source Data for Expanded View [file EMBR-18-0-s003.zip › SDataEV/Figure_EV2_CXXXD_original_source_dataXXX.pdf]

Figure 2E & 2F

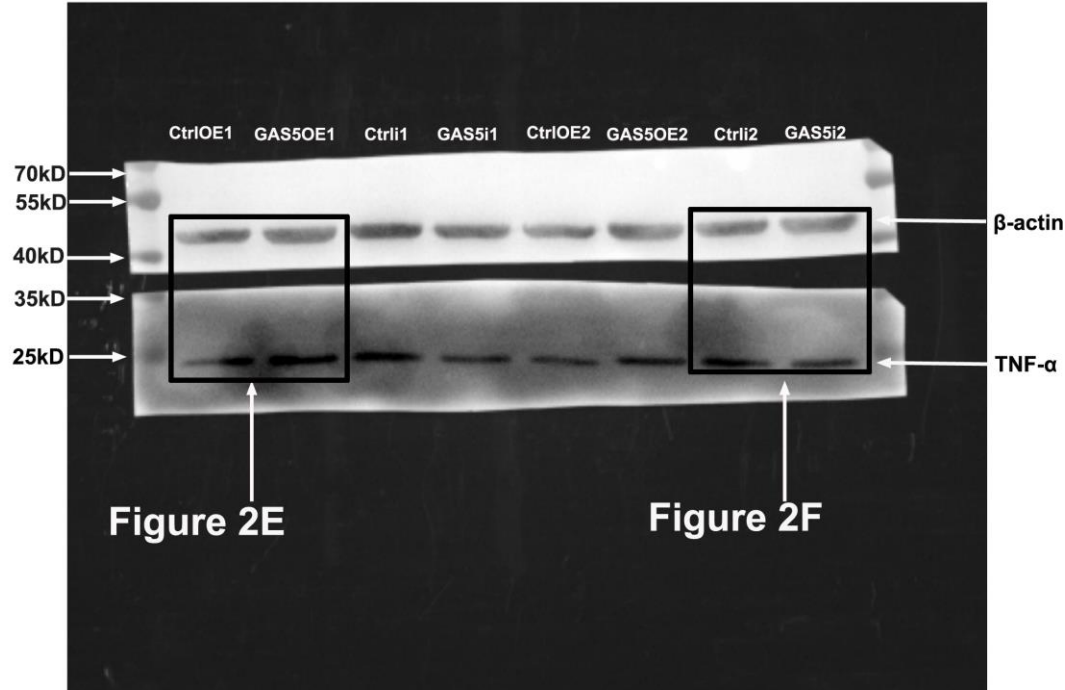

Supplement: Supplementary file 5 — Source Data for Figure 2 [file EMBR-18-0-s004.pdf]

**Figure 7B**

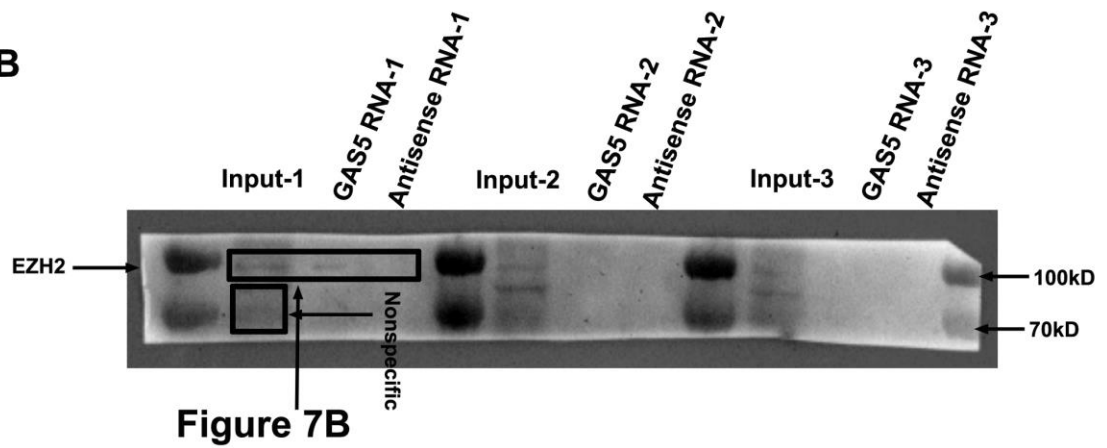

**Figure 7K**

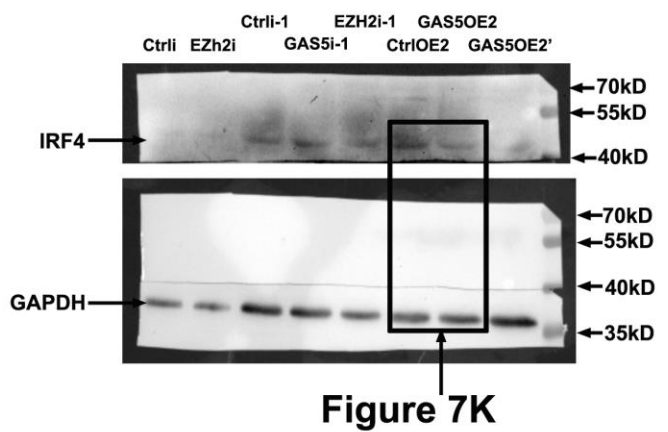

**Figure 7L**

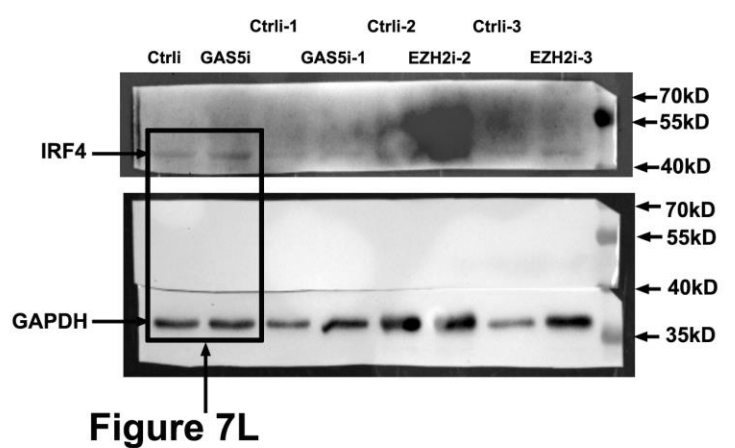

**Figure 7M**

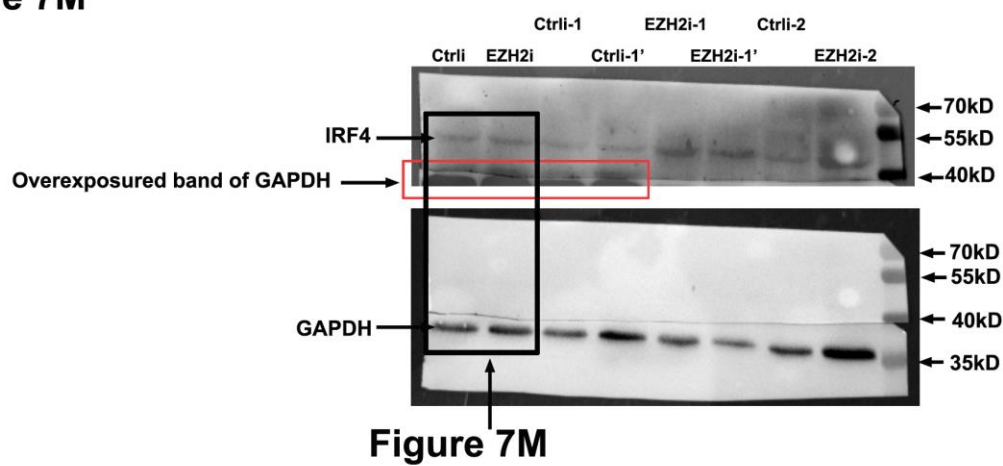

Supplement: Supplementary file 6 — Source Data for Figure 7 [file EMBR-18-0-s005.pdf]
